# Psychosocial Factors Influencing Individual Well-Being in Chinese Adolescents in Hong Kong: a Six-Year Longitudinal Study

**DOI:** 10.1007/s11482-017-9545-4

**Published:** 2017-06-23

**Authors:** Daniel T. L. Shek, Lu-Yin Liang

**Affiliations:** 1 Department of Applied Social Sciences, The Hong Kong Polytechnic University, Hong Kong, China; 2 Department of Social Work, Guangdong University of Technology, Guangzhou, China

**Keywords:** Subjective well-being, Life satisfaction, Hopelessness, Adolescent

## Abstract

This pioneer study investigated the longitudinal development of adolescent subjective well-being (SWB) in terms of life satisfaction and hopelessness. The concurrent and longitudinal influence of different socio-demographic characteristics (i.e., age, gender, economic disadvantage, and family intactness), individual qualities (i.e., resilience, social competence, positive identity, and spirituality), and familial characteristics (i.e., family functioning, and parent-child relationship) on these two aspects of SWB were examined. A total of 3328 Hong Kong students from 28 secondary schools participated in a 6-year longitudinal study. While adolescent life satisfaction showed a declining trend, hopelessness gradually increased across the six years. Resilience, social competence, family functioning, and father-child relational qualities were significant predictors of life satisfaction at the initial status, whereas gender, mother-child relational qualities, positive identity and spirituality predicted changes in life satisfaction over time. Regarding hopelessness, gender, family intactness, resilience, social competence, father-child relational qualities, and mother-child relational qualities were significant correlates at the initial slope, but spirituality and family functioning were the longitudinal predictors of hopelessness over the adolescence period. While the present study showed that some existing Western findings can be replicated in the Chinese context, there are some novel and puzzling observations deserving further scrutiny.

## Introduction

Subjective well-being (SWB) is a term commonly used to describe healthy and successful individual functioning, positive social relationships, and social ecology, provides safety, human and civil rights, social justice and participation in civil society (Andrews et al. 2002). It is widely considered as the primary goal and the general concern of most people (Diener 1998). The connotation of SWB covers many domains in a person’s life, including the resources, strengths, problems and needs. According to Diener (1984), the structure of SWB could be conceptualized as consisting of two interrelated and distinctive components - cognitive component and emotional component. It is defined as the overall assessment of attitudes and feelings regarding one’s life at a particular point in time ranging from negative to positive evaluation (Diener 1984). Life satisfaction has been commonly measured as a salient cognitive appraisal of SWB and quality of life in adolescence (Diener and Diener 1995; Diener et al. 1985; Diener et al. 1999).

On the other hand, hopelessness, which constitutes a system of negative beliefs and expectancies concerning oneself and one’s future, is regarded as a key emotional component of SWB (Shek 2008). The existing studies have found that life satisfaction was negatively associated with hopelessness in the Western (Gilman and Huebner 2003; Heisel and Flett 2004; Kolarcik et al. 2012; Proctor et al. 2009) and Chinese samples (Shek 2005, 2007a).

With specific reference to adolescents, their SWB is highly volatile as they are in a crucial stage characterized by significant changes in different developmental domains (Eccles 1999). Besides, research shows that SWB may have a major effect on positive youth outcomes for making a smooth transition to adulthood (Eccles et al. 2008). Over the past decades, there has been a growing body of adolescent research that underscored and highlighted the importance of SWB in adolescent development.

Unfortunately, there are several limitations in the related studies in this field. First, although many studies have been conducted to assess adolescent life satisfaction and hopelessness, very few of them have attempted to measure these two components in a single study. Obviously, inclusion of both life satisfaction and hopelessness in a single study can help researchers understand SWB in adolescents in a holistic manner. Second, although many studies have examined personal influences of life satisfaction and hopelessness (Abdollahi and Talib 2015; Gooding et al. 2012; Hjemdal et al. 2012; Johnson et al. 2010; Mo et al. 2014), few studies have attempted to understand how family well-being might influence adolescent life satisfaction and hopelessness. Third, there are few studies examining the differential contribution of personal and familial factors to adolescent life satisfaction. Fourth, as most of the existing studies are cross-sectional studies, there is a great need to conduct longitudinal studies in this area. Fifth, there are few studies in non-Western contexts. To tackle the above problems, this study attempted to investigate changes of life satisfaction and hopelessness amongst adolescents in a Chinese context using longitudinal data. In this study, the influences of personal factors (socio-demographic factors and positive youth development attributes) and familial factors on the developmental variations of these two constructs were examined.

### SWB in Adolescents

Life satisfaction is commonly regarded as an individual’s cognitive evaluation of the quality of life based on his or her own judgment (Diener et al. 1985). It acts as a core construct of positive psychology that focuses on identifying strengths and buffers against the development of psychopathological issues (Veenhoven 1988). The theory of needs describes life satisfaction as a pivotal psychological variable which explains how well the basic needs are satisfied and how well other goals are considered as attainable (Cummins and Nistico 2002). According to this view, adolescents who are able to accomplish different positive development goals and who are satisfied with the fulfillment of different needs may exhibit higher levels of satisfaction with life. Existing research revealed that a high level of life satisfaction was positively related to physical health, social relationship, and academic performance in adolescence (Diener et al. 2002; Kim et al. 2013; Miller and Thoresen 2003) but negatively related to mental health problems such as depression and suicidal ideation (Goldbeck et al. 2007). Besides, adolescent life satisfaction is a protective factor of externalizing behavior in adolescents, including delinquent behavior, aggression and victimization, problematic Internet use, substance abuse, and sexual behavior (Desousa et al. 2008; Martin et al. 2008; McKnight et al. 2002; Piko et al. 2005; Proctor et al. 2009; Senol-Durak and Durak 2011; Suldo and Huebner 2004a; Valois et al. 2006; Zullig et al. 2001). Life satisfaction also enhances the adaptive functioning and coping strength, which could reduce susceptibility to stress and risks (Sun and Shek 2013).

Since adolescence is a difficult phase in human development with many changes, life satisfaction throughout this transition period may not be static (Arnett 1999). Interestingly, although the importance of life satisfaction in adolescents is widely suggested, there are not many related empirical studies and the available findings are consistent. On the one hand, studies showed that high school students generally had moderately high levels of life satisfaction (McCullough et al. 2000) and life satisfaction in adolescence was stable (Ash and Huebner 2001; Casas et al. 2007; Suldo and Huebner 2004b). On the other hand, studies showed that adolescent life satisfaction gradually decreased across the adolescent period (Goldbeck et al. 2007; Michel et al. 2009). In view of the conflicting research findings, more work in this area is needed.

Hopelessness refers to the pessimistic perceptions and expectancies regarding oneself and one’s capacities, one’s future, and prospects for the world around them (Beck et al. 1974; Ostrander et al. 1995; Prociuk et al. 1976). An adolescent who feels hopeless tends to hold the beliefs that terrible outcomes will occur and that nothing could be changed for better. The theory of hopelessness describes the mediating role of hopelessness in the relationship between cognitive vulnerability and psychological problems such as depression. According to this theory, the occurrence of negative life events evokes the presence of cognitive vulnerability, with both of them mutually interacting and further contributing to the formation of hopelessness, which eventually triggers depression. Empirically, many studies showed that hopelessness was the strongest cognitive predictor in the concurrent relationship with suicidal ideation (Cukrowicz et al. 2004; Rutter and Behrendt 2004; Warman et al. 2004) and hopelessness was a key risk factor for different types of problem behavior, such as substance abuse (Bolland 2003; Bolland et al. 2001; DuRant et al. 1994). On the contrary, hopelessness was negatively linked with academic achievement and goal attainment (Curry et al. 1997; Snyder 2002; Snyder et al. 1991).

While the impacts of hopelessness on adolescent development have received increasing attention, only a few studies have measured its changes throughout the adolescent transition period. Stoddard, Henly, Sieving, and Bolland (2011) assessed the hopelessness trajectory among 723 African Americans and found that there was an upward trend in the development of hopelessness in adolescence. This observation is consistent with the longitudinal findings of Shek and Li (2016)’s study based on 2427 students in Hong Kong. In contrast, Lester (2015) reported that hopelessness of American adolescents did not undergo any significant variation. In view of the inconsistent findings, there is still a need to conduct more longitudinal studies to clarify the developmental trend of adolescent hopelessness.

### Predictors of SWB in Adolescents

Although studies have been conducted to examine the antecedents of SWB in adolescents, the evidence is basically inconclusive. With regard to the socio-demographic factors, mixed findings were observed for the effect of gender on adolescent perceived life satisfaction and hopelessness. For example, while Goldbeck et al. (2007) found that boys were more satisfied with life compared to girls, several studies have reported contrary results (Al-Attiyah and Nasser 2016; Huebner 1997; Huebner et al. 2000). Similarly, although Duke, Borowsky, Pettingell, and McMorris (2011) claimed that girls showed a moderately high level of hopelessness, some studies showed that hopelessness was higher in boys (Lester 2015; Siyahhan et al. 2012). Regarding age, while some studies did not show age effect on subjective well-being in adolescents (Al-Attiyah and Nasser 2016; Bolland 2003), Duke, Borowsky, Pettingell, and McMorris (2011) reported that age predicted adolescent life satisfaction. Besides, there is fragmented evidence on the influence of family attributes on adolescent subjective well-being. While some studies showed that family intactness was a significant predictor of life satisfaction and hopelessness (Antaramian et al. 2008; Duke et al. 2011; Lai-Kwok and Shek 2010), the role of economic disadvantage on adolescent life satisfaction and hopelessness is inconclusive (Bradley and Corwyn 2004; Grob et al. 1996).

Besides background socio-demographic correlates, studies showed that positive youth development attributes were related to the SWB of adolescents. Research showed that adolescents with higher levels of resilience (Gooding et al. 2012; Hjemdal et al. 2012; Johnson et al. 2010; Mo et al. 2014; Sun and Shek 2010) and spirituality (Abdollahi and Talib 2015; Khan et al. 2011) showed higher life satisfaction and lower hopelessness. Similarly, social competence (Ciarrochi et al. 2003) and positive identity (Johnson et al. 2010; Sun and Shek 2010, 2013) were related to adolescent subjective well-being.

Compared with individual factors, there are relatively fewer studies on the relationships between family well-being and adolescent subjective well-being. According to the Circumplex Model, cohesion is one of the central dimensions of marital and family system (Olson et al. 2014). “*Jia he wan shi xing*” (a harmonious family will prosper) is an old Chinese saying that highlights the importance of harmony and the absence of conflicts in the Chinese family relationship. Many global and local research findings have suggested that healthy family functioning played an essential role in influencing the well-being of adolescents (Leung & Shek, 2016; Leung, Shek & Lin, 2016). An increase in family functioning was found to be related to an increase in life satisfaction and a decrease in hopelessness (Lai-Kwok and Shek 2008; Raboteg-Šarić et al. 2009; Sun and Shek 2013). Research also shows that parent-child relational qualities could serve as a buffer against hopelessness and a protective factor of life satisfaction in adolescence (Lai-Kwok and Shek 2010; Leung and Zhang 2000; Stoddard et al. 2011).

Although many studies have examined the predictors of life satisfaction and hopelessness in adolescents, most of them collected data at one point in time only, which is unable to help the researchers understand the changes of adolescent subjective well-being over time. Besides, few studies ever comprehensively examined both personal and familial factors associated with life satisfaction and hopelessness. There is a general lack of sufficient and consistent findings on the role of socio-demographic factors as well.

In view of the above-mentioned research gaps, this study intended to address three questions as follows:

- *Research Question 1:* What are the developmental trends of adolescent subjective well-being indexed by life satisfaction and hopelessness in the high school years? Based on the existing studies (Goldbeck et al. 2007; Michel et al. 2009; Shek and Li 2016), it was predicted that there would be a drop in adolescent life satisfaction (Hypothesis 1) and a rise in adolescent hopelessness (Hypothesis 2).
- *Research Question 2:* Are socio-demographic factors (age and gender), structural family attributes (family intactness and economic disadvantage), positive youth development attributes (resilience, psychosocial competence, positive identity and spirituality) and family processes (family functioning and parent-child relational qualities) related to the initial level (Question 2a) and changes (Question 2b) of adolescent life satisfaction? With specific reference to the positive youth development (PYD) attributes, the general prediction was that PYD attributes would be positively related to the initial level of life satisfaction (Hypothesis 3) and its changes (Hypothesis 4). For family processes, it was predicted that family attributes would be positively related to the initial level and changes of life satisfaction (Hypotheses 5 and 6).
- *Research Question 3:* Are socio-demographic factors (age and gender), structural family attributes (family intactness and economic disadvantage), positive youth development attributes (resilience, psychosocial competence, positive identity and spirituality) and family processes (family functioning and parent-child relational qualities) related to the initial level (Question 3a) and changes (Question 3b) of hopelessness in adolescents? Based on the literature, it was expected that PYD attributes would be positively related to the initial level and changes of hopelessness (Hypotheses 7 and 8). For family processes, it was predicted that family attributes would be positively related to the initial level and changes of hopelessness (Hypotheses 9 and 10).

## Methods

### Participants

In this study, data were collected from 28 government-funded secondary schools in Hong Kong. A total of 3328 Hong Kong students were invited to participate in a six-year longitudinal study which attempted to assess the developmental trend of SWB of adolescents (M age = 12.59, SD = 0.74 years). The data were collected six times between 2009/2010 and 2014/2015 school years. As the participants are minors, parental consent was obtained prior to data collection. Although some students were absent, dropped out or transferred to other schools, the attrition rates across the six waves were lower than 30%, which is acceptable for a longitudinal study (Taris 2000). Overall speaking, the gender ratio of the participants was roughly equal across waves. As seen in Table 1, the percentage of participants who experienced family financial hardship ranged from 4.6% to 6.8%, and about 15.5% to 18.1% of the participants came from a non-intact family (Table 1).

**Table 1:** Profile of participants

	Wave 1	%	Wave 2^a^	%	Wave 3^a^	%	Wave 4^a^	%	Wave 5^a^	%	Wave 6^a^	%
N (Participants)	3328		2905		2860		2684		2474		2385	
Gender												
Male	1719	51.7	1445	49.7	1433	50.1	1336	49.8	1200	48.5	1161	48.7
Female	1572	47.2	1419	48.8	1407	49.2	1338	49.9	1265	51.1	1218	51.1
Economic disadvantage												
NOT receiving CSSA^b^	2606	78.3	2377	81.8	2341	81.9	2269	84.5	2131	86.1	2063	86.5
Receiving CSSA	225	6.8	160	5.5	147	5.1	132	4.9	114	4.6	110	4.6
Family intactness												
Intact families	2781	83.6	2415	83.1	2397	83.8	2213	82.5	2027	81.9	1948	81.7
Non-intact families	515	15.5	469	16.1	455	15.9	466	17.4	441	17.8	432	18.1

^a^the numbers were based on the participants who ever participated in Wave 1 assessment, as only those joining Wave 1 assessment were included in LMM. The numbers of the students who did not report the corresponding information are not presented

^b^denotes Comprehensive Social Security Assistance Scheme

### Measures

#### Satisfaction with Life Scale

Developed by Diener, Emmons, Larsen, and Griffin (1985), life satisfaction scale is a five-item instrument that intends to assess the respondents’ own satisfaction with life. The translated version of this scale was used in this study. The reliability and validity of the scale have been supported by previous research (Shek 1998). It has also shown good internal consistency in this study as the alpha values ranged from 0.851 to 0.885 across the six waves.

#### Hopelessness Scale

The respondents’ perceptions of their level of hopelessness were measured by a modified five-item scale which was developed by Beck and his colleagues (Beck et al. 1974). The scale has been shown to have good reliability and validity in the previous study (Shek 1993). In this study, the alpha values of the measure ranged from 0.854 to 0.895 across waves.

#### Chinese Positive Youth Development Scale

This is a 90-item scale developed for measuring several aspects of positive youth development. The scale consists of 15 subscales, and four of them were used for assessing the resilience (6 items), social competence (7 items), clear and positive identity (7 items), and spirituality (7 items) in this study. With the alpha values ranging from 0.896 to 0.911 across the six waves, this measure has shown satisfactory internal consistency.

#### Chinese Family Assessment Instrument

A nine-item instrument was adopted for assessing family functioning in a Chinese context with proven reliability and validity. This scale measures three dimensions of family functioning, including mutuality, conflicts, and communication. The values of Cronbach’s alpha ranged from 0.900 to 0.916 across the six waves, which showed good reliability.

#### Father-Child Subsystem Quality and Mother-Child Subsystem Quality Measures

The quality of father-child relationship was measured by the father-child subsystem quality scale, which was developed by Shek (2006, 2007b). Similar items were also applied in the measurement of the quality of mother-child relationship. Several aspects of father/mother-child relationship quality were examined in this measure, including paternal/maternal knowledge, paternal/maternal expectation, paternal/maternal monitoring, paternal/maternal psychological control, children’s satisfaction with paternal/maternal control and father/mother-child communication. The reliability for the subscale of father-child relationship ranged from 0.871 to 0.885 across waves. The subscale of mother-child relationship also showed good internal consistency as its Cronbach’s alpha values across waves were from 0.880 to 0.887. The previous studies have shown that the related measures possessed good psychometric properties (Shek and Law 2016).

#### Demographic Information

In this study, four items were designed for measuring the socio-demographic characteristics, including age, gender, family intactness, and economic disadvantage.

### Data Analytic Plan

In the present study, the developmental trajectories of life satisfaction, hopelessness and their related predictors were examined by the individual growth curve modeling (IGC) approach, which is regarded as one of the most effective strategies in estimating the intra- and inter-individual differences over time. Under this approach, a two-level hierarchical model that nests time within individual was applied (Shek and Ma 2011). On the first level, three unconditional growth models including unconditional initial model (Model 1), unconditional linear model (Model 2), and unconditional quadratic model (Model 3) were employed for estimating the intra-individual conditions of life satisfaction and hopelessness in the initial stage and their changes over time. On the second level, the conditional model (Model 4) was constructed for estimating how the individual growth parameters contained at Level 1 were associated with a series of between-subject socio-demographic predictors (i.e., age, gender, family intactness, and economic disadvantage) and other relevant factors (i.e., family attributes, positive youth development attributes, and family process factors). The model fit was examined by −2 log likelihood, the Akaike Information Criterion (AIC), and the Bayesian Information Criterion (BIC). The lower values indicate a better fit of the model.

Based on the findings from the IGC analysis, prototypical plots were generated for demonstrating and interpreting the growth trend of life satisfaction and hopelessness and their interactions with different predictors across time. The procedures described in Shek and Ma (2011) were applied for the generation of the plots:

- Level 1:Y_ij_ = β_oj_ + β_1j_ (Time) + β_2j_ (Time^2^) + r_ij_
- Level 2: β_0j_ = r_00_ + r_01_(age) + r_02_(gender) + r_03_(family intactness) + r_04_(economic disadvantage) + r_05_(family attributes) + r_06_(positive youth development attributes) + r_07_(family process factors)
- *β*_*1j*_ = *r*_*10*_ *+ r*_*11*_(age) *+ r*_*12*_(gender) *+ r*_*13*_(family intactness) *+ r*_*14*_(economic disadvantage) *+ r*_*15*_(family attributes) *+ r*_*16*_(positive youth development attributes) *+ r*_*17*_(family process factors)
- *β*_*2j*_ = *r*_*20*_ *+ r*_*21*_(age) *+ r*_*22*_(gender) *+ r*_*23*_(family intactness) *+ r*_*24*_(economic disadvantage) *+ r*_*25*_(family attributes) *+ r*_*26*_(positive youth development attributes) *+ r*_*27*_(family process factors)

## Results

**Fig. 1:**
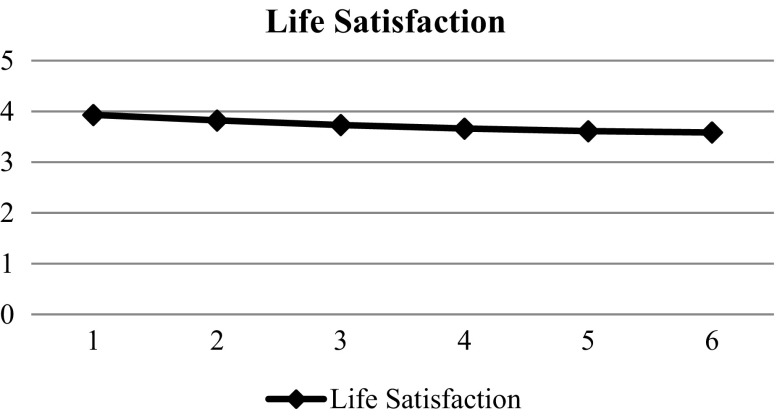
Prototypical trajectories of life satisfaction across six waves (all samples)

Correlation analyses showed that all the measured factors were significantly associated with life satisfaction and hopelessness (Tables 2 and 3). As shown in Table 4, the quadratic model (Model 3) fitted the data significantly better than Model 1 (Δχ2 (7) = 1163.316, *p* < 0.001; ΔAIC = 1149.316; ΔBIC = 1093.372) and Model 2 (Δχ2 (4) = 123.501, *p* < 0.001; ΔAIC = 115.501; ΔBIC = 83.533). Significant and positive values in intercept (β = 3.929, SE = 0.18, *p* < 0.01), linear (β = −0.120, SE = 0.12, *p* < 0.01), and quadratic effects (β = 0.009, SE = 0.002, *p* < 0.01) were found in Model 3, suggesting that life satisfaction decreased across the six waves and the declining rate gradually slowed down (Fig. 1). The findings provided support for Hypothesis 1.

**Table 2:** Correlation among variables (life satisfaction)

Variables	1.	2.	3.	4.	5.	6.	7.	8.	9.	10.	11.	12.	13.	14.	15.	16.	17.
1. LS	1																
2. SLS	.552^**^	1															
3. TLS	.483^**^	.581^**^	1														
4. FLS	.425^**^	.491^**^	.591^**^	1													
5. GLS	.416^**^	.476^**^	.561^**^	.636^**^	1												
6. QLS	.375^**^	.436^**^	.517^**^	.571^**^	.663^**^	1											
7. Age	-.061^**^	-.042^**^	-.046^**^	-.009	.019^*^	.016	1										
8. Gender	-.007	-.030^**^	.025^**^	.062^**^	.042^**^	.045^**^	-.030^**^	1									
9. Family Intactness	.096^**^	.090^**^	.049^**^	.054^**^	.051^**^	.052^**^	-.064^**^	-.008	1								
10. Economic Disadvantage	-.047^**^	-.082^**^	-.072^**^	-.067^**^	-.084^**^	-.068^**^	-.020^*^	.067^**^	-.125^**^	1							
11. Resilience	.450^**^	.315^**^	.273^**^	.260^**^	.244^**^	.216^**^	.013	-.020^*^	.048^**^	-.002	1						
12. Psychosocial Competence	.384^**^	.274^**^	.243^**^	.235^**^	.200^**^	.178^**^	.000	.067^**^	.062^**^	.017^*^	.479^**^	1					
13. Positive Identity	.460^**^	.308^**^	.267^**^	.243^**^	.222^**^	.213^**^	-.011	.079^**^	.063^**^	.013	.495^**^	.510^**^	1				
14. Spirituality	.608^**^	.436^**^	.373^**^	.323^**^	.316^**^	.277^**^	-.038^**^	.041^**^	.086^**^	-.004	.533^**^	.454^**^	.514^**^	1			
15. Family Functioning	.538^**^	.420^**^	.348^**^	.289^**^	.300^**^	.265^**^	-.078^**^	-.049^**^	.184^**^	-.003	.384^**^	.347^**^	.387^**^	.499^**^	1		
16. Father-child Relationship Qualities	.450^**^	.349^**^	.299^**^	.261^**^	.264^**^	.238^**^	-.053^**^	.015	.196^**^	-.033^**^	.322^**^	.286^**^	.354^**^	.413^**^	.611^**^	1	
17. Mother-child Relationship Qualities	.417^**^	.306^**^	.247^**^	.191^**^	.195^**^	.169^**^	-.083^**^	.070^**^	.110^**^	.032^**^	.337^**^	.281^**^	.334^**^	.410^**^	.600^**^	.484^**^	1

*Correlation is significant at the 0.05 level (2-tailed)

**Correlation is significant at the 0.01 level (2-tailed)

**Table 3:** Correlation among variables (hopelessness)

Variables	1.	2.	3.	4.	5.	6.	7.	8.	9.	10.	11.	12.	13.	14.	15.	16.	17.
1. HL	1																
2. SHL	.451^**^	1															
3. THL	.404^**^	.536^**^	1														
4. FHL	.345^**^	.406^**^	.538^**^	1													
5. GHL	.337^**^	.410^**^	.512^**^	.584^**^	1												
6. QHL	.325^**^	.383^**^	.464^**^	.515^**^	.621^**^	1											
7. Age	.027^**^	-.006	.022^**^	.012	.003	-.029^**^	1										
8. Gender	-.064^**^	-.044^**^	-.069^**^	-.092^**^	-.106^**^	-.103^**^	-.030^**^	1									
9. Family Intactness	-.029^**^	-.054^**^	-.039^**^	-.031^**^	-.032^**^	-.047^**^	-.064^**^	-.008	1								
10. Economic Disadvantage	-.058^**^	-.008	-.016^*^	-.014	-.022^**^	-.004	-.020^*^	.067^**^	.125^**^	1							
11. Resilience	-.375^**^	-.313^**^	-.286^**^	-.267^**^	-.267^**^	-.259^**^	.013	-.020^*^	.048^**^	-.002	1						
12. Psychosocial Competence	-.292^**^	-.277^**^	-.231^**^	-.222^**^	-.234^**^	-.212^**^	.000	.067^**^	.062^**^	.017^*^	.479^**^	1					
13. Positive Identity	-.305^**^	-.263^**^	-.245^**^	-.247^**^	-.255^**^	-.271^**^	-.011	.079^**^	.063^**^	.013	.495^**^	.510^**^	1				
14. Spirituality	-.469^**^	-.350^**^	-.331^**^	-.277^**^	-.284^**^	-.289^**^	-.038^**^	.041^**^	.086^**^	-.004	.533^**^	.454^**^	.514^**^	1			
15. Family Functioning	-.403^**^	-.318^**^	-.278^**^	-.231^**^	-.242^**^	-.248^**^	-.078^**^	-.049^**^	.184^**^	-.003	.384^**^	.347^**^	.387^**^	.499^**^	1		
16. Father-child Relationship Qualities	-.330^**^	-.293^**^	-.262^**^	-.237^**^	-.220^**^	-.228^**^	-.053^**^	.015	.196^**^	-.033^**^	.322^**^	.286^**^	.354^**^	.413^**^	.611^**^	1	
17. Mother-child Relationship Qualities	-.345^**^	-.283^**^	-.243^**^	-.219^**^	-.211^**^	-.203^**^	-.083^**^	.070^**^	.110^**^	.032^**^	.337^**^	.281^**^	.334^**^	.410^**^	.600^**^	.484^**^	1

**Table 4:** Results of unconditional growth models (life satisfaction)

		Model 1		Model 2		Model 3	
		Estimate	SE	Estimate	SE	Estimate	SE
Fixed effects							
Intercept	*β*_*0j*_						
Intercept	*γ*_*00*_	3.713***	.012	3.896***	.016	3.929***	.018
Linear Slope	*β*_*1j*_						
Time	*γ*_*10*_			-.075***	.004	-.120***	.012
Quadratic Slope	*β*_*2j*_						
Time^2^	*γ*_*20*_					.009***	.002
Random effects							
Level 1 (within)							
Residual	*r*_*ij*_	.565***	.006	.470***	.006	.443***	.006
Level 2 (between)							
Intercept	*u*_*0j*_	.622***	.016	.785***	.025	.810***	.030
Time	*u*_*1j*_			-.065***	.005	-.124***	.017
Time^2^	*u*_*2j*_					.146***	.015
Fit statistics							
Deviance		58,099.191		57,059.376		56,935.875	
AIC		58,105.191		57,071.376		56,955.875	
BIC		58,129.167		57,119.328		57,035.795	
df		3		6		10	

Model 1 = unconditional mean model; model 2 = unconditional linear growth model; model 3 = unconditional quadratic growth model

*** *p* < .001

**Fig. 2:**
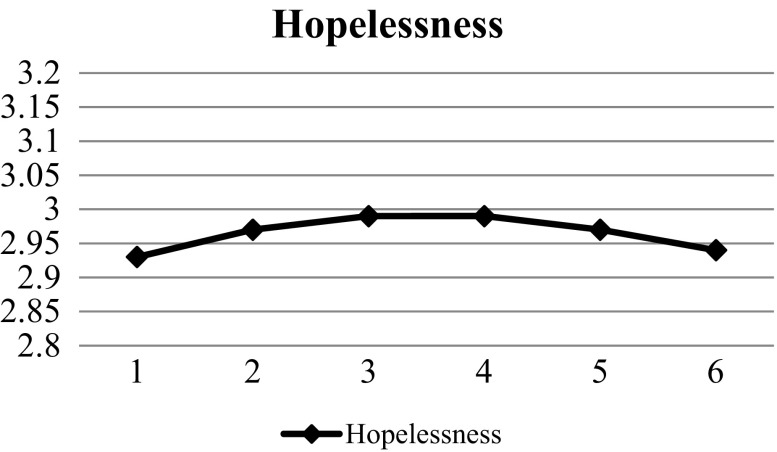
Prototypical trajectories of hopelessness across six waves (all samples)

Regarding hopelessness, the findings in Table 5 indicated that Model 3 had the best model fit as compared with Model 1(Δχ2 (7) = 528.996, *p* < 0.001; ΔAIC = 514.996; ΔBIC = 459.075) and Model 2 (Δχ2 (4) = 147.147, *p* < 0.001; ΔAIC = 139.147; ΔBIC = 107.193). Moreover, significant results were observed in intercept (β = 2.730, SE = 0.19, *p* < 0.01), linear slope (β = 0.048, SE = 0.14, *p* < 0.01) and quadratic slope (β = −0.009, SE = 0.003, *p* < 0.01) in Model 3, implying that hopelessness increased constantly but the increasing rate slowed down over time (Fig. 2). The findings provided support for Hypothesis 2.

**Table 5:** Results of unconditional growth models (hopelessness)

		Model 1		Model 2		Model 3	
		Estimate	SE	Estimate	SE	Estimate	SE
Fixed effects							
Intercept	*β*_*0j*_						
Intercept	*γ*_*00*_	2.775***	.013	2.762***	.017	2.730***	.019
Linear Slope	*β*_*1j*_						
Time	*γ*_*10*_			.005	.004	.048***	.014
Quadratic Slope	*β*_*2j*_						
Time^2^	*γ*_*20*_					-.009***	.003
Random effects							
Level 1 (within)							
Residual	*r*_*ij*_	.685***	.008	.600***	.008	.564***	.008
Level 2 (between)							
Intercept	*u*_*0j*_	.626***	.017	.801***	.028	.847***	.034
Time	*u*_*1j*_			.030***	.002	.197***	.020
Time^2^	*u*_*2j*_					.005***	.001
Fit statistics							
Deviance		61,346.789		60,964.940		60,817.793	
AIC		61,352.789		60,976.940		60,837.793	
BIC		61,376.754		61,024.872		60,917.679	
df		3		6		10	

Model 1 = unconditional mean model; model 2 = unconditional linear growth model; model 3 = unconditional quadratic growth model

*** *p* < .001

**Fig. 3:**
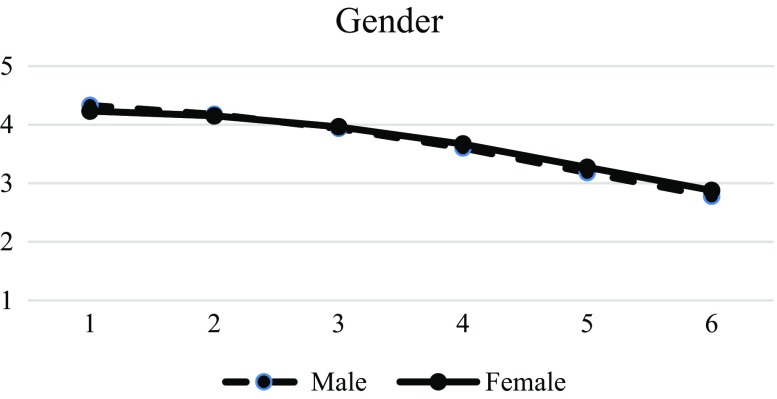
Prototypical trajectories of life satisfaction in male and female students across six waves

**Fig. 4:**
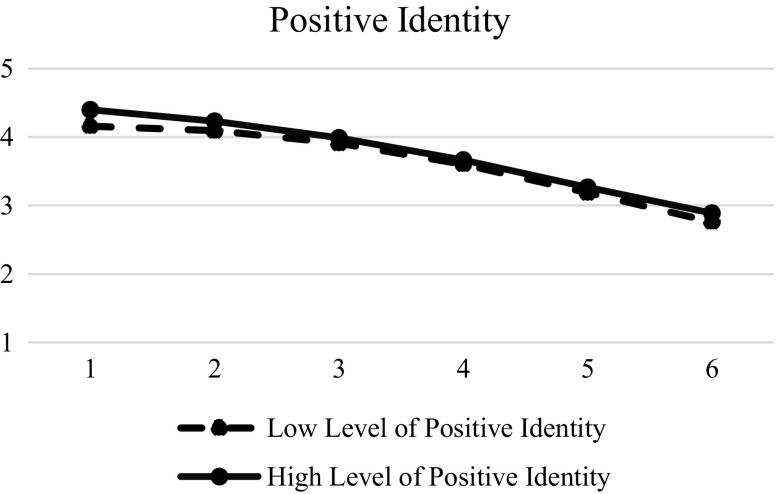
Prototypical trajectories of life satisfaction in students with high level of positive identity and low level of positive identity across six waves

**Fig. 5:**
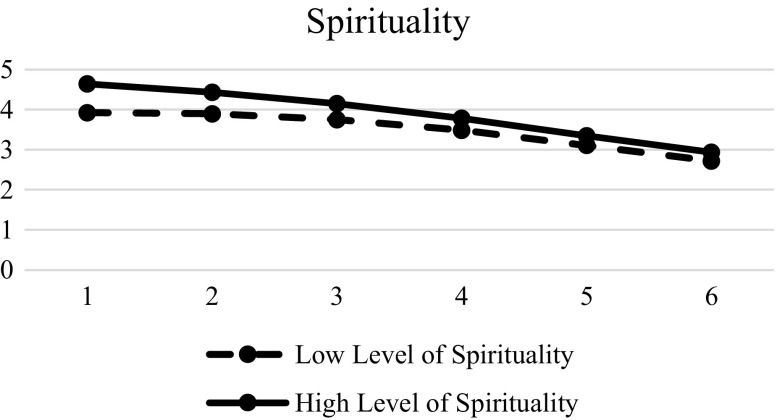
Prototypical trajectories of life satisfaction in students with high level of spirituality and low level of spirituality across six waves

Table 6 presents the IGC findings on the different predictors of life satisfaction. Results showed that gender was a significant factor in the initial status (β = 0.053, SE = 0.015, *p* < 0.01) and linear slope (β = −0.036, SE = 0.014, *p* < 0.01). According to Fig. 3, male adolescents displayed a higher level of life satisfaction in the initial assessment, but had a faster declining rate than female adolescents. Positive identity and spirituality were significant predictors of initial status (positive identity: β = 0.116, SE = 0.019, *p* < 0.01; spirituality: β = 0.361, SE = 0.019, *p* < 0.01), linear slope (positive identity: β = 0.212, SE = 0.018, *p* < 0.01; spirituality: β = −0.011, SE = 0.018, *p* < 0.01), and quadratic slope (positive identity: β = 0.009, SE = 0.003, *p* < 0.01; spirituality: β = 0.011, SE = 0.004, *p* < 0.01) of life satisfaction. In the initial assessment, adolescents with higher positive identity and spirituality showed a higher level of life satisfaction. However, life satisfaction for the adolescents with higher positive identity or spirituality dropped faster than those with lower positive identity or spirituality (Figs. 4 and 5).

**Table 6:** Results of LMM models with level-2 predictors (life satisfaction)

		Model 4	
		Estimate	SE
Fixed effects			
Intercept	*β*_*0j*_		
Intercept	*γ*_00_	4.228***	.253
Age	*γ*_01_	-.024	.020
Gender ^a^	*γ*_02_	-.053***	.015
Family Intactness	*γ*_03_	-.002	.022
Economic Disadvantage	*γ*_04_	-.057	.031
Resilience	*γ*_05_	.061***	.018
Psychosocial Competence	*γ*_06_	.046**	.018
Positive Identity	*γ*_07_	.116***	.019
Spirituality	*γ*_08_	.361***	.019
Family Functioning	*γ*_09_	.212***	.022
Father-child Relationship Qualities	*γ*_010_	.102***	.019
Mother-child Relationship Qualities	*γ*_011_	.050**	.019
Linear slope	*β*_*1j*_		
Intercept	*γ*_10_	-.084	.239
Age	*γ*_11_	-.004	.020
Gender ^a^	*γ*_12_	.036**	.014
Family Intactness	*γ*_13_	-.024	.021
Economic Disadvantage	*γ*_14_	-.030	.029
Resilience	*γ*_15_	-.009	.017
Psychosocial Competence	*γ*_16_	.009	.017
Positive Identity	*γ*_17_	-.055**	.018
Spirituality	*γ*_18_	-.101***	.018
Family Functioning	*γ*_19_	-.036	.020
Father-child Relationship Qualities	*γ*_110_	-.002	.018
Mother-child Relationship Qualities	*γ*_111_	-.042*	.017
Quadratic slope	*β*_2j_		
Intercept	γ_20_	-.052	.048
Age	*γ*_21_	.005	.004
Gender ^a^	*γ*_22_	-.004	.003
Family Intactness	*γ*_23_	.004	.004
Economic Disadvantage	*γ*_24_	.002	.006
Resilience	*γ*_25_	.001	.003
Psychosocial Competence	*γ*_26_	-.002	.003
Positive Identity	*γ*_27_	.009*	.003
Spirituality	*γ*_28_	.011**	.004
Family Functioning	*γ*_29_	.005	.004
Father-child Relationship Qualities	*γ*_210_	.0002	.003
Mother-child Relationship Qualities	*γ*_211_	.005	.003
Random effects			
Level 1 (within)			
Residual	*r*_*ij*_	.433	.007
Level 2 (between)			
Intercept	*u*_*0j*_	.234	.017
Time	*u*_*1j*_	.108	.015
Time^2^	*u*_*2j*_	.003	.001
Fit statistics			
Deviance		35,648.016	
AIC		35,734.016	
BIC		36,060.589	
df		43	

*** *p* < .001; ** *p* < .01, * *p* < .05

^a^ Male = 1, Female = −1

**Fig. 6:**
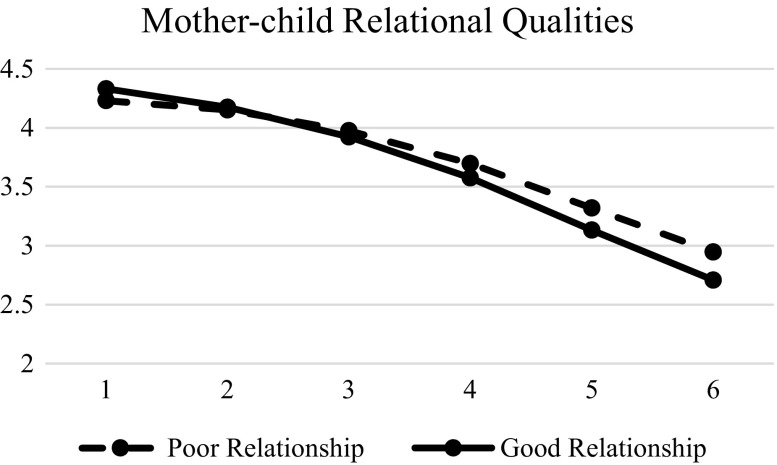
Prototypical trajectories of life satisfaction in students with good mother-child relationship and poor mother-child relationship across six waves

Resilience (β = 0.061, SE = 0.018, *p* < 0.01), social competence (β = 0.046, SE = 0.018, *p* < 0.01), family functioning (β = 0.212, SE = 0.022, *p* < 0.01), and father-child relational qualities (β = 0.102, SE = 0.19, *p* < 0.01) were significant predictors of initial status. Besides, mother-child relational quality was significant in initial status (β = 0.050, SE = 0.019, *p* < 0.01) and linear change (β = −0.042, SE = 0.017, *p* < 0.01). In other words, adolescents with a good relationship with mother displayed more life satisfaction than those who had a poor mother-child relationship in the initial assessment, but had a faster decreasing rate over time (Fig. 6). The support for Hypotheses 3 to 6 are presented in Table 8. Basically, there was mixed support for these hypotheses.

**Fig. 7:**
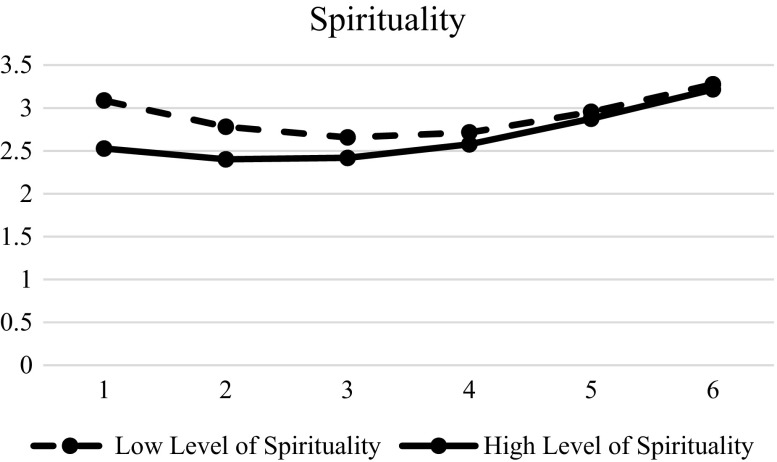
Prototypical trajectories of hopelessness in students with high level of spirituality and low level of spirituality across six waves

**Fig. 8:**
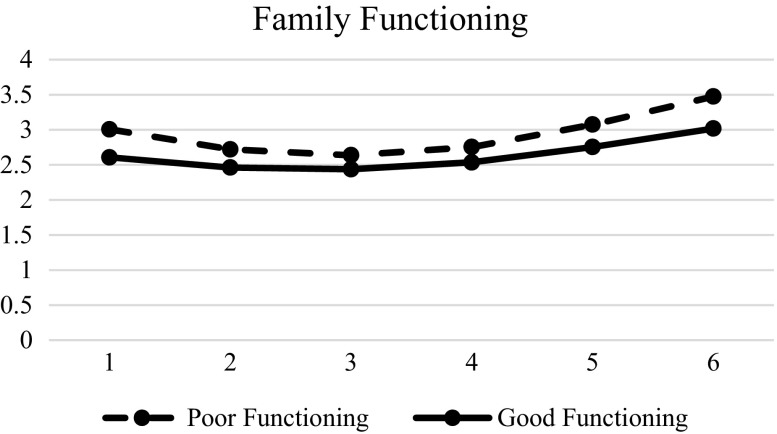
Prototypical trajectories of hopelessness in students with good family functioning and poor family functioning across six waves

Table 7 shows the effects of different predictors of the inter-individual change on hopelessness. Results showed that gender (β = −0.002, SE = 0.016, *p* < 0.01), family intactness (β = 0.055, SE = 0.027, *p* < 0.01), resilience (β = −0.124, SE = 0.022, *p* < 0.01), social competence (β = −0.044, SE = 0.022, *p* < 0.01), father-child relational qualities (β = −0.065, SE = 0.023, *p* < 0.01), and mother-child relational qualities (β = −0.079, SE = 0.023, *p* < 0.01) were significant in the initial status. Spirituality was a significant predictor of initial status (β = −0.282, SE = 0.024, *p* < 0.01), linear slope (β = 0.095, SE = 0.021, *p* < 0.01) and quadratic slopes (β = −0.013, SE = 0.004, *p* < 0.01), suggesting that the adolescents with lower spirituality had higher hopelessness in the beginning, while the adolescents with higher spirituality would increase hopelessness more (Fig. 7). Similarly, family functioning was also significant at the initial status (β = −0.196, SE = 0.026, *p* < 0.01), linear slope (β = 0.093, SE = 0.023, *p* < 0.01), and quadratic slope (β = −0.015, SE = 0.004, *p* < 0.01). Adolescents with poorer family functioning showed higher hopelessness in the beginning, but adolescents with better family functioning would increase hopelessness faster (Fig. 8). The support for Hypotheses 7 to 10 are presented in Table 8. Basically, there was mixed support for these hypotheses.

**Table 7:** Results of LMM models with level-2 predictors (hopelessness)

		Model 4	
		Estimate	SE
Fixed effects			
Intercept	*β*_*0j*_		
Intercept	*γ*_00_	2.727***	.309
Age	*γ*_01_	-.012	.024
Gender ^a^	*γ*_02_	-.002**	.016
Family Intactness	*γ*_03_	.055*	.027
Economic Disadvantage	*γ*_04_	-.044	.038
Resilience	*γ*_05_	-.124 ***	.022
Psychosocial Competence	*γ*_06_	-.044*	.022
Positive Identity	*γ*_07_	.021	.023
Spirituality	*γ*_08_	-.282***	.024
Family Functioning	*γ*_09_	-.196***	.026
Father-child Relationship Qualities	*γ*_010_	-.065**	.023
Mother-child Relationship Qualities	*γ*_011_	-.079***	.023
Linear slope	*β*_*1j*_		
Intercept	*γ*_10_	-.231	.275
Age	*γ*_11_	.027	.022
Gender ^a^	*γ*_12_	-.057	.018
Family Intactness	*γ*_13_	-.016	.024
Economic Disadvantage	*γ*_14_	.063	.033
Resilience	*γ*_15_	.001	.020
Psychosocial Competence	*γ*_16_	-.018	.019
Positive Identity	*γ*_17_	-.010	.020
Spirituality	*γ*_18_	.095***	.021
Family Functioning	*γ*_19_	.093***	.023
Father-child Relationship Qualities	*γ*_110_	-.033	.020
Mother-child Relationship Qualities	*γ*_111_	.006	.020
Quadratic slope	*β*_2j_		
Intercept	γ_20_	.068	.053
Age	*γ*_21_	-.007	.004
Gender ^a^	*γ*_22_	-.002	.003
Family Intactness	*γ*_23_	.003	.005
Economic Disadvantage	*γ*_24_	-.010	.006
Resilience	*γ*_25_	.001	.004
Psychosocial Competence	*γ*_26_	.005	.004
Positive Identity	*γ*_27_	-.003	.004
Spirituality	*γ*_28_	-.013***	.004
Family Functioning	*γ*_29_	-.015***	.004
Father-child Relationship Qualities	*γ*_210_	.007	.004
Mother-child Relationship Qualities	*γ*_211_	.002	.004
Random effects			
Level 1 (within)			
Residual	*r*_*ij*_	.537	.009
Level 2 (between)			
Intercept	*u*_*0j*_	.443	.025
Time	*u*_*1j*_	.178	.020
Time^2^	*u*_*2j*_	.004	.001
Fit statistics			
Deviance		38,789.197	
AIC		38,875.197	
BIC		39,201.723	
df		43	

*** *p* < .001; ** *p* < .01, * *p* < .05

^a^ Male = 1, Female = −1

**Table 8:** Summary of the hypotheses tested

Hypothesis	Variable	Findings (Support: √; Not support: X; Odd finding:!)		
		Initial Status	Linear Change	Quadratic Change
**H1:** There would be a drop in adolescent life satisfaction.	Life satisfaction	√		
**H2:** There would be an increase in adolescent hopelessness.	Hopelessness	√		
**H3-H4:** PYD attributes would be positively related to the initial level and change of life satisfaction.	Resilience	√	X	X
	Psychosocial competence	√	X	X
	Positive identity	√	!	!
	Spirituality	√	!	!
**H5-H6:** Family attributes would be positively related to the initial level and change of life satisfaction.	Family functioning	√	X	X
	Father-child relational qualities	√	X	X
	Mother-child relational qualities	√	!	X
**H7-H8:** PYD attributes would be positively related to the initial level and change of hopelessness.	Resilience	√	X	X
	Psychosocial competence	√	X	X
	Positive identity	X	X	X
	Spirituality	√	!	!
**H9-H10:** Family attributes would be positively related to the initial level and change of hopelessness.	Family functioning	√	!	!
	Father-child relational qualities	√	X	X
	Mother-child relational qualities	√	X	X

## Discussion

Overall speaking, this research has achieved several breakthroughs in understanding the developmental trajectories of adolescent well-being indexed by life satisfaction and hopelessness. Fundamentally, it examined the longitudinal effects of several predictors on these two aspects of adolescent well-being. Regarding methodological advancement, individual growth curve modeling was used, which helps to examine the change and rate of change in adolescent SWB. A large sample size was also adopted in the study, which helps to improve the generalizability of the findings. Finally, this is the first scientific longitudinal study which examined the well-being of adolescents over the high school years in different Chinese contexts.

Consistent with some previous literature, this study found that adolescent life satisfaction exhibited a decreasing trend, while their hopelessness level was increasing (Michel et al. 2009; Shek and Li 2016), thus providing support for Hypotheses 1 and 2. Adolescents’ decreased life satisfaction and increased hopelessness could be explained by the confusion and the developmental challenges they face during the transitional process, notably the increase in studying pressure as well as future career decisions and problems engendered by peers or dating (Erol and Ergun 2013; Goldbeck et al. 2007; Shek and Li 2016). Theoretically, the specific changes that shape well-being in the adult years deserve further investigation.

This study underscores the role of different psychosocial factors associated with adolescent life satisfaction and hopelessness. For life satisfaction, gender was the only socio-demographic predictor that displayed significant concurrent and long-term effects. According to the findings, life satisfaction in males decreased faster than that of females. It is possible that males’ late maturation and females’ early maturation may contribute to the change of their life satisfaction. Besides, all PYD factors (resilience, psychosocial competence, positive identity and spirituality) and familial factors (family functioning, father-child relational qualities and mother-child relational qualities) significantly predicted the initial level of life satisfaction as expected. These findings are generally consistent with the results of the existing research (e.g., Al-Attiyah and Nasser 2016; Huebner et al. 2000; Lester 2015; Siyahhan et al. 2012). However, those who had higher PYD scores (positive identity and spirituality) and mother-child relational qualities actually had a slower growth rate in life satisfaction, although those with higher related scores had higher life satisfaction level than did those with lower related scores throughout the adolescent period. There are three possible explanations for these odd findings. First, ceiling effect of life satisfaction scores may account for the findings. That is, for those with better PYD and mother-child relational qualities, their life satisfaction may be already high, thus resulting in a relatively slower growth rate for life satisfaction. Second, those with high positive identity, spirituality and/or mother-child relational qualities may have higher expectations about life, hence reducing their satisfaction with life. Third, for mother-child relational qualities, the finding may be due to the possibility that maternal over-control or over-protection constrains adolescents’ decision-making autonomy and limits their exposure to responsibilities and opportunities, which lead to their increased risk of maladjustment for late adolescence. The impact may be more pronounced in the Chinese families where there is a strong emphasis on parental control (Shek and Sun, 2014). These plausible explanations deserve further studies in future.

The current findings also showed some significant predictors for hopelessness. Regarding socio-demographic correlates, the significant gender effect at initial status which indicated that female adolescents had higher level of hopelessness than male adolescents is not in line with the findings in some existing research (Lester 2015; Siyahhan et al. 2012). This is also not entirely consistent with the observation that adolescent boys were less satisfied than adolescent girls. While the significant effect of family intactness was also reported in previous studies, age and economic disadvantage were not predictors for hopelessness. As predicted, all PYD factors and family attributes significantly predicted the initial status, with the exception of positive identity. These findings are consistent with the existing research findings (Abdollahi and Talib 2015; Gooding et al. 2012; Lai-Kwok and Shek 2008).

However, the effects of spirituality and family functioning on the rates of change are more puzzling. Similar to life satisfaction, it may be the consequence of floor effect (i.e., those who have high PYD or family functioning may have a lower starting level of hopelessness). Apart from this possibility, although some adolescents reported a higher level of spirituality, it does not mean they are mature enough to have full understanding of the real purpose in life at this stage (i.e., moratorium with commitment but without confusion). Although finding goals in life (such as striving for academic excellence) may be meaningful, this may also trigger confusion, uncertainty and frustration. This process is not a smooth ride, especially when it mixes with different challenges derived from academic, vocational and social pursuits. Thus, young people with higher level of spirituality in late adolescence may think more and experience more struggles, which ultimately leads to the elevated level of hopelessness. Similarly, good family functioning tends to bring high expectations for young people. As such, adolescents with better family functioning may be slow in attaining their ideal life. Obviously, future studies should be conducted to examine these possibilities surrounding these puzzles.

There are some limitations of this study that need to be further addressed. First, this study examined both personal and familial factors on adolescents’ SWB. However, as school is also a key environment influencing adolescent development, it would be exciting to examine school factors such as academic achievement, peer relationship, and the teacher-student relationship as well. Second, as personal competences and qualities were reported by the students solely, it would be great if data could be collected from the significant-others such as parents, teachers and social workers. Last but not least, due to different cultural contexts, adolescents in the West may not have the same family experiences and personal developments as compared with Chinese adolescents. Hence, their development of SWB may be different as well. Therefore, it would be exciting to conduct a comparative study to examine differences between the Western and Chinese adolescents for observing the related cultural differences. Despite these limitations, this pioneer study has promoted our understanding of the development of adolescent well-being (life satisfaction and hopelessness) and the related psychosocial determinants (socio-demographic factors including gender, age and structural family characteristics, positive youth development attributes and family well-being) which provide the lead for further studies.
